# Supplementary material for: Analysis of Aedes aegypti microRNAs in response to Wolbachia wAlbB infection and their potential role in mosquito longevity
Source: Sci Rep. 2022 Sep 9;12:15245. doi: 10.1038/s41598-022-19574-x (PMC9463151; doi:10.1038/s41598-022-19574-x)
Supplement: Supplementary file 1 — Supplementary Information. [file 41598_2022_19574_MOESM1_ESM.pdf]

Analysis of *Aedes aegypti* microRNAs in response to *Wolbachia w*AlbB infection and their potential role in mosquito longevity

Cameron Bishop, Mazhar Hussain, Leon E. Hugo, Sassan Asgari

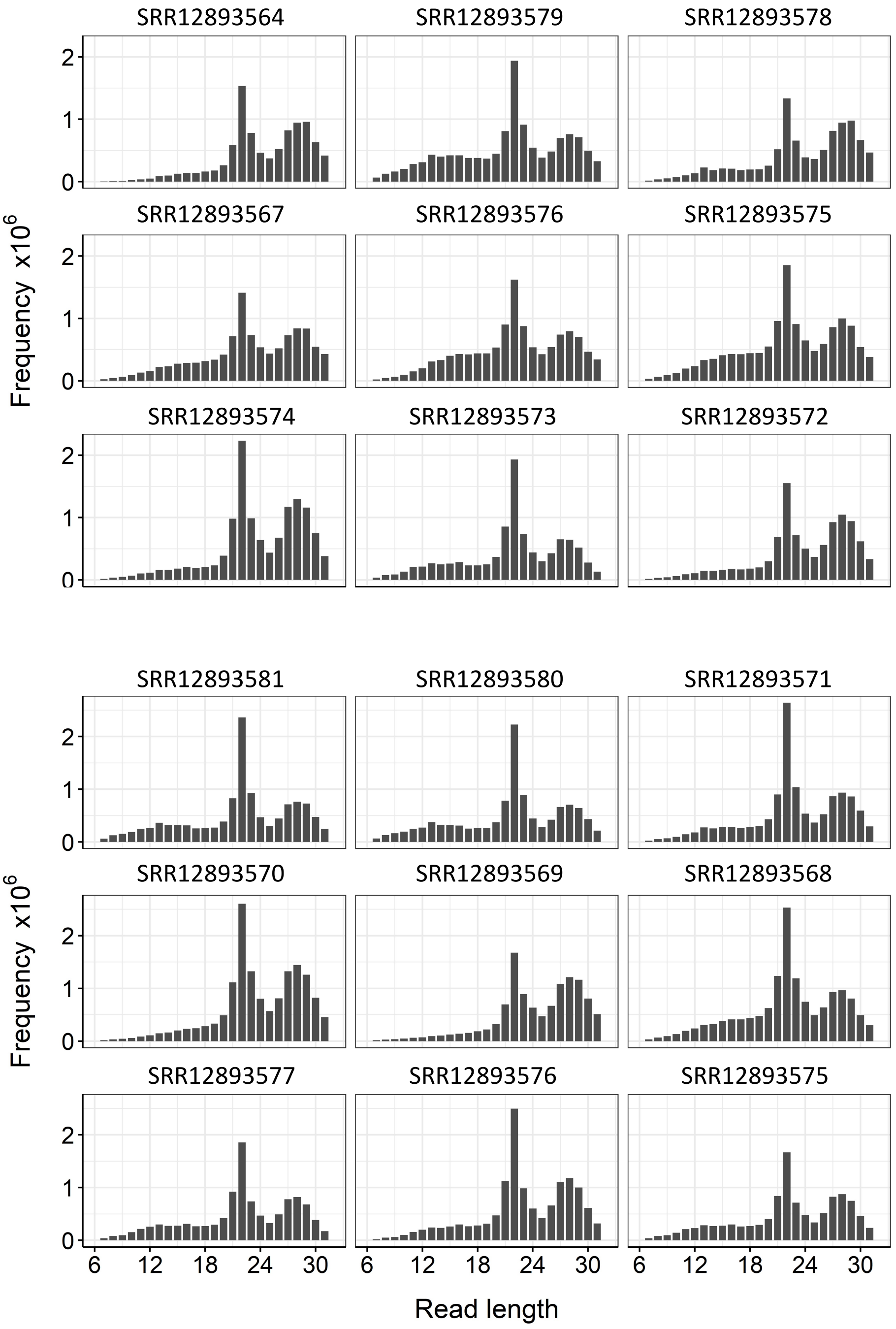

Figure S1: Read-length distribution of small RNA-Seq libraries.

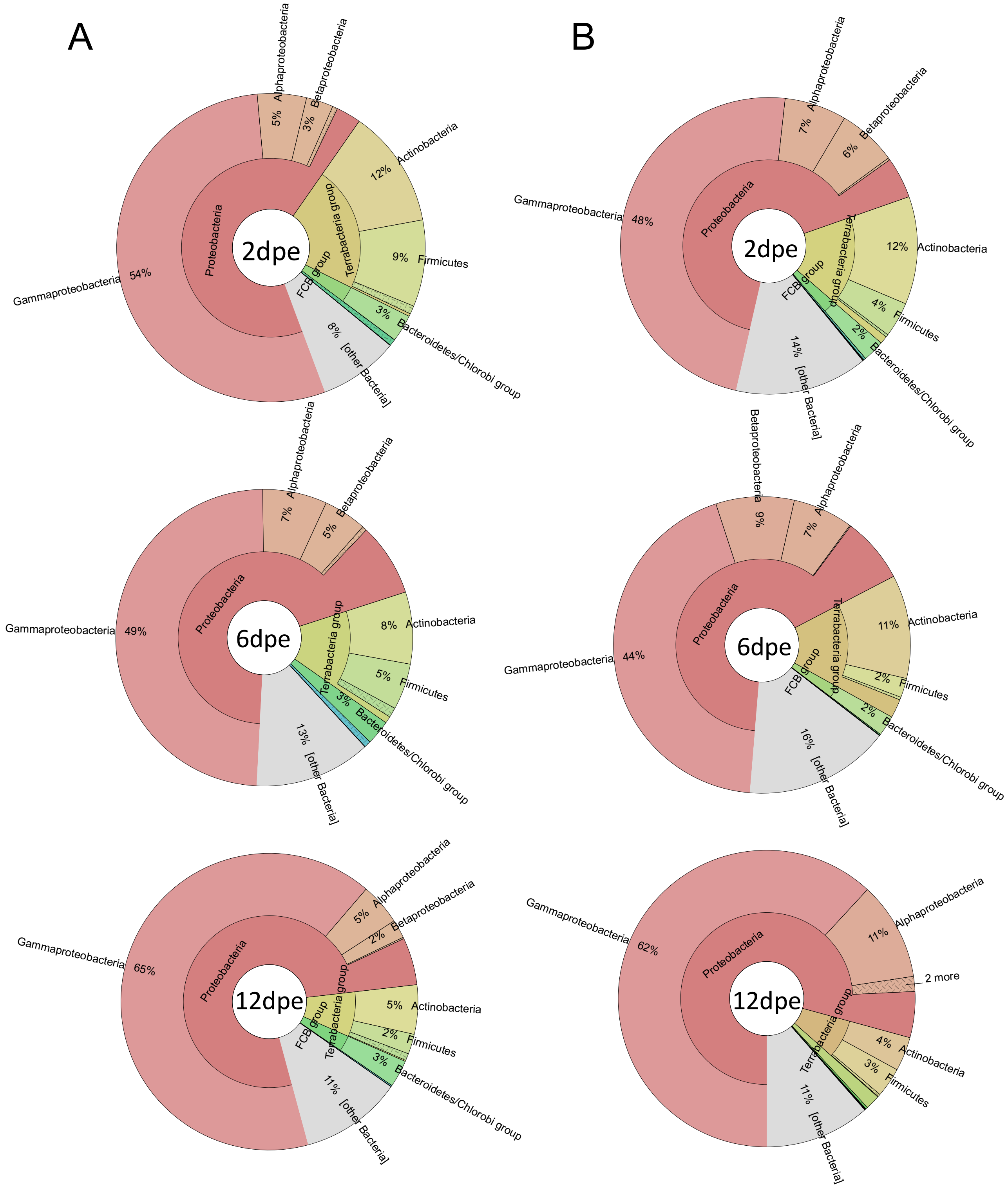

Figure S2: Bacterial community composition is consistent between WB2 and WB2.tet mosquitoes. Results of Kraken analysis of reads that did not map to either **A)** *Ae. aegypti* or *w*AlbB in WB2 mosquitoes, or **B)** *Ae. aegypti* in WB2.tet mosquitoes.

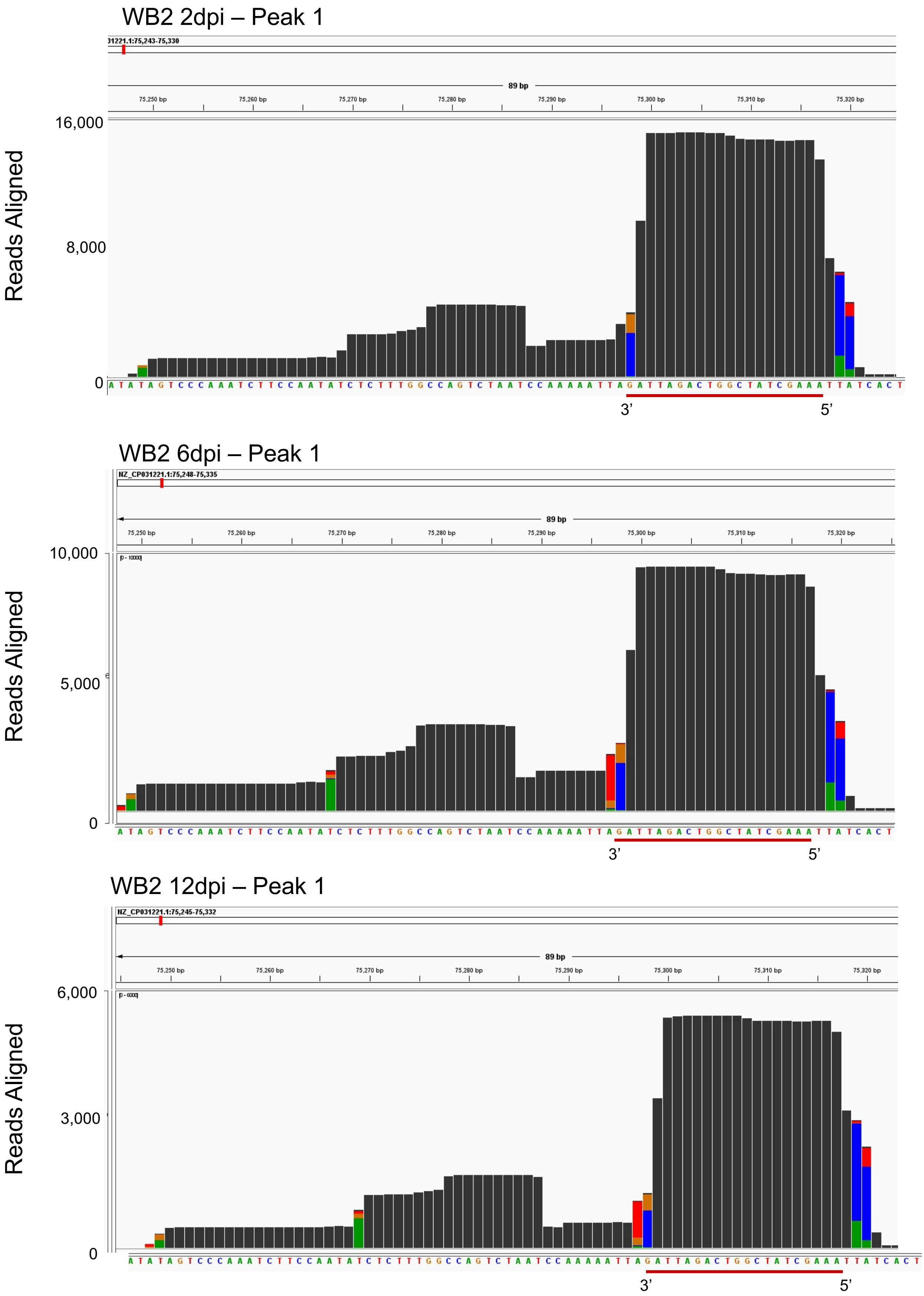

Figure S3: Per-base small RNA-SEQ read depth of the predicted hairpin corresponding to Peak1.

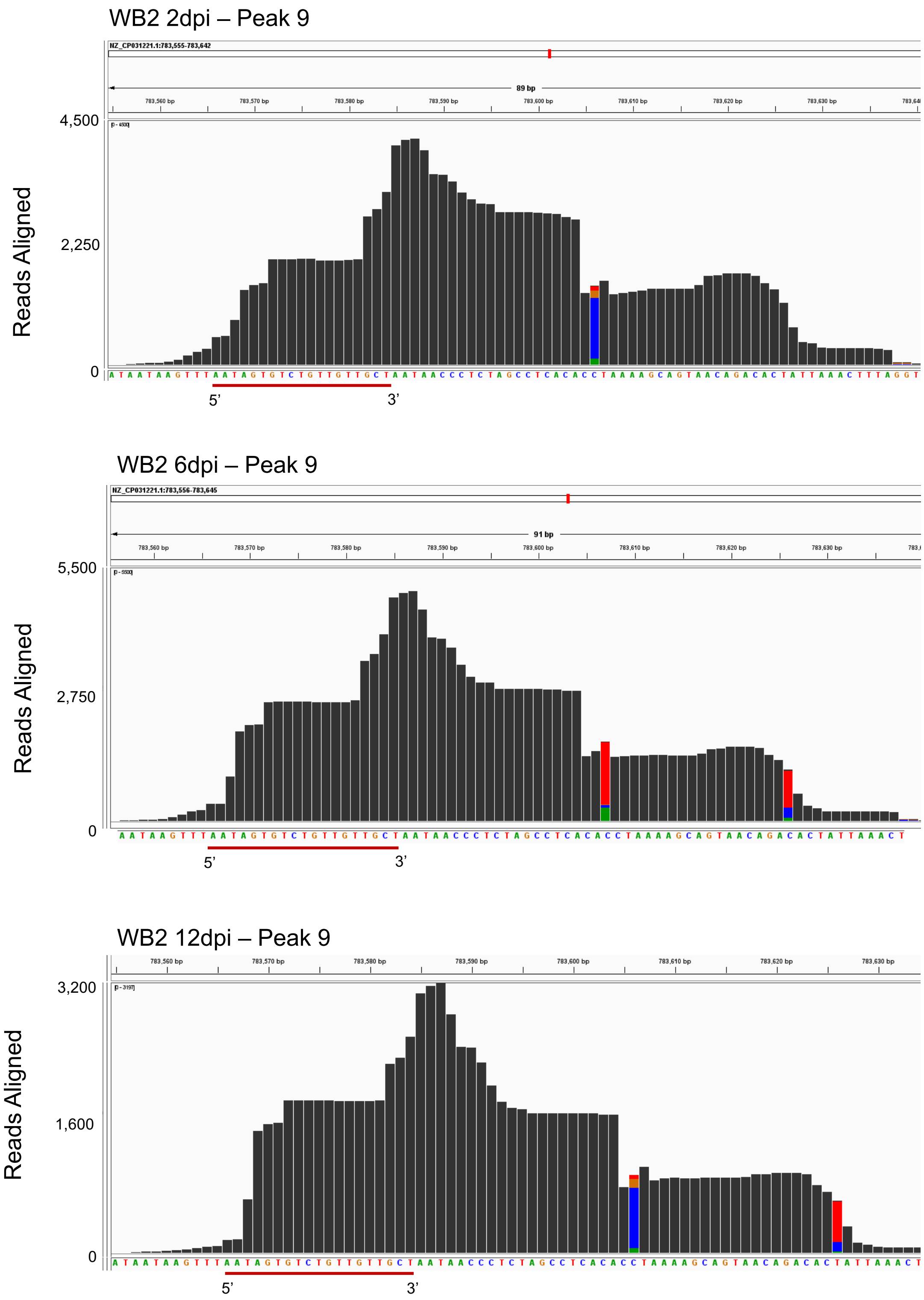

Figure S4: Per-base small RNA-SEQ read depth of the predicted hairpin corresponding to Peak 9.

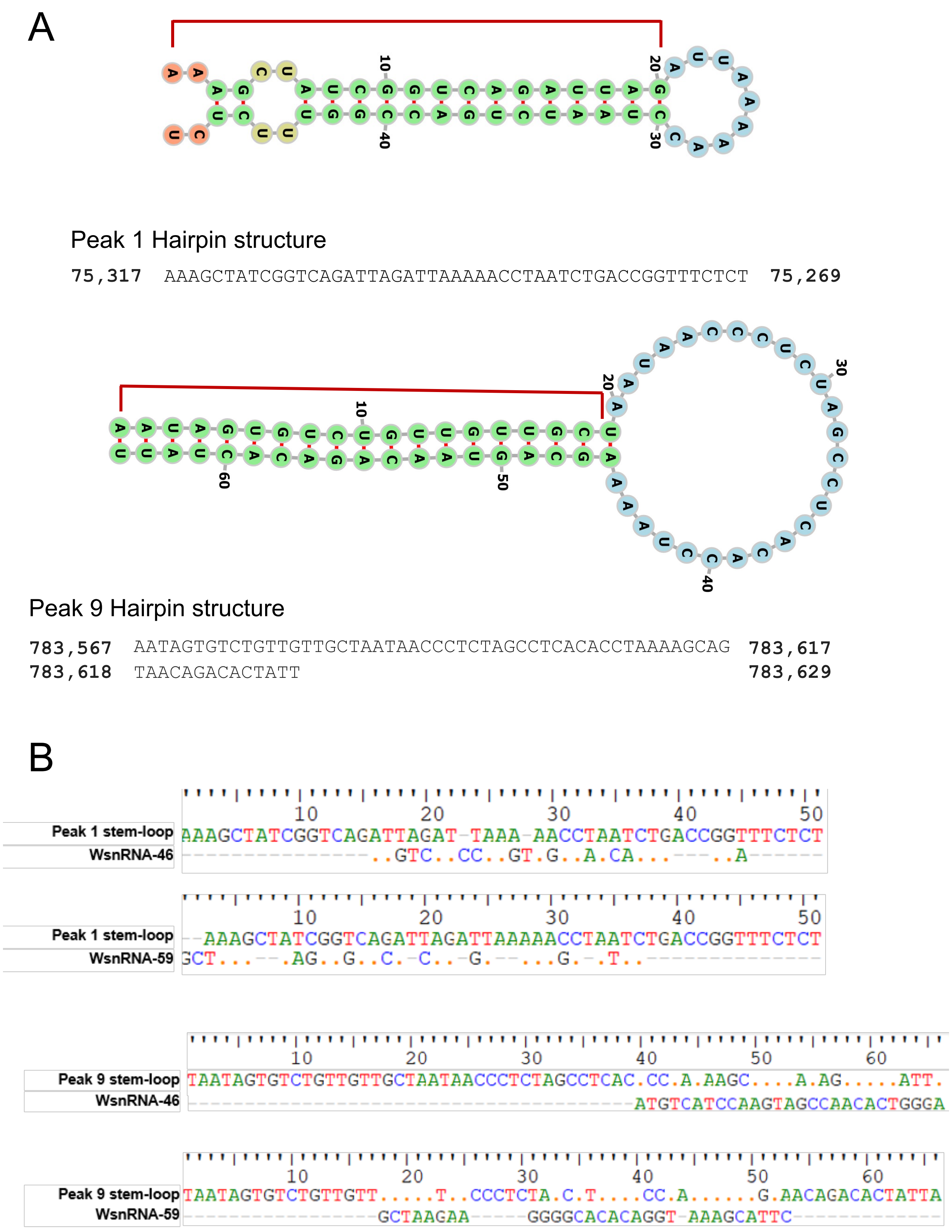

Figure S5: *w*AlbB-derived miRNA-like small RNAs. (A) Predicted hairpin structures corresponding to two regions of the *w*AlbB NZ_CP031221.1 genome that had high coverage of small RNA-Seq reads in libraries derived from WB2 mosquitos. Genomic coordinates indicate the start and end positions of the putative hairpin structure, identified by two peaks of 18-24nt long small RNA-Seq reads. **(B)** Sequence alignment between each predicted stem-loop structure and *W*snRNA-46 and *W*snRNA-59 from Mayoral et al., 2014. Dots represent homologous positions.

Table S1. Primers and oligos used in this study.

Primer/oligo	sequence
wsp qF	ATCTTTTATAGCTGGTGGTGGT
wsp qR	GGAGTGATAGGCATATCTTCAAT
Rps17 qF	CACTCCGAGGTCCGTGGTAT
Rps17 qR	GGACACTTCGGGCACGTAGT
Ae. aegypti 5s ribosomal RNA	CGCGTCAGAATGTGAACT
AAEL010793 qF	CCTACGACTTCGATTGGGAACA
AAEL010793 qR	ATCGTATGCGATCGTACGCC
AAEL006171 qF	CTGGCGTAGTTGAAAAGATG
AAEL006171 qR	ATGTCAGCAAACCTTGGCCC
AAEL010508 qF	ACGAAAATCGCACGGATGGA
AAEL010508 qR	CGTTGGGCTTGTAAGTGGTG
AAEL006095 qF	CGGCGATTCGTACATCGTCT
AAEL006095 qR	CAGCTGGACGCTGAGGATAG
AAEL006113 qF	GGGCATCGATCTTTGGGGAT
AAEL006113 qR	CGTAGTCAGCGTGGTTGTCT
AAEL003402 qF	TATTGTGCGACGCCAAGAGC
AAEL003402 qR	GCCCGAAAGGGCATAGGATA
miRNA-190-5p inhibitor	AGAUAUGUUUGAUAUUCUUGGUU
miRNA-276b-5p inhibitor	AGCGAGGUAUAGAGUUCCUAU
Negative control inhibitor	CAGUACUUUUGUGUAGUACAA

Table S2: Small RNA-Seq QC mapping statistics.

SRR number	Sample name	Raw reads	Passed QC	18-24nt	Mapped x1	reads mpd >1	total mapped	% mapped (18-24nt)
SRR12893564	WB2.tet 2dpe	13,927,840	13,926,548	3,970,511	2,094,977	1,736,845	3,831,822	96.51%
SRR12893579	WB2.tet 2dpe	15,457,915	15,441,355	5,405,159	2,582,994	2,631,028	5,214,022	96.46%
SRR12893578	WB2.tet 2dpe	15,519,324	15,514,368	3,552,681	1,774,756	1,641,041	3,415,797	96.15%
SRR12893577	WB2.tet 6dpe	15,264,515	15,258,858	4,473,178	2,240,998	2,017,260	4,258,258	95.20%
SRR12893576	WB2.tet 6dpe	14,370,561	14,368,661	5,347,725	2,748,098	2,372,449	5,120,547	95.75%
SRR12893575	WB2.tet 6dpe	16,493,955	16,488,519	5,805,852	3,004,578	2,552,044	5,556,622	95.71%
SRR12893574	WB2.tet 12dpe	17,180,689	17,167,215	5,671,283	3,336,000	2,119,813	5,455,813	96.20%
SRR12893573	WB2.tet 12dpe	10,499,400	10,487,552	4,817,700	2,745,585	1,899,599	4,645,184	96.42%
SRR12893572	WB2.tet 12dpe	14,454,900	14,445,830	4,135,898	2,341,992	1,623,159	3,965,151	95.87%
SRR12893581	WB2 2dpe	13,975,176	13,945,588	5,515,965	2,930,216	2,192,051	5,122,267	92.86%
SRR12893580	WB2 2dpe	12,853,978	12,835,587	5,248,114	2,786,821	2,093,335	4,880,156	92.99%
SRR12893571	WB2 2dpe	14,962,000	14,952,181	6,130,774	3,179,142	2,556,910	5,736,052	93.56%
SRR12893570	WB2 6dpe	17,981,052	17,970,746	6,950,626	3,665,646	2,845,681	6,511,327	93.68%
SRR12893569	WB2 6dpe	16,037,719	16,025,114	4,623,920	2,396,939	1,870,612	4,267,551	92.29%
SRR12893568	WB2 6dpe	16,675,896	16,662,294	7,250,301	3,706,494	2,946,139	6,652,633	91.76%
SRR12893567	WB2 12dpe	11,859,587	11,833,863	4,962,521	2,649,644	1,906,861	4,556,505	91.82%
SRR12893566	WB2 12dpe	15,539,404	15,526,359	6,272,518	3,506,929	2,353,557	5,860,486	93.43%
SRR12893565	WB2 12dpe	12,698,221	12,670,765	4,664,429	2,464,867	1,818,082	4,282,949	91.82%

Table S3: Genomic coordinates of precursor and mature miRNA loci in the AaeL5.0 reference genome. The base mean is the mean of counts of all samples, normalized for sequencing depth.

miRNA	structure	Chromosome	Start	End	Base mean
aae-miR-33	precursor	NC_035107.1	50,888,874	50,888,954	--
aae-miR-33-3p	mature	NC_035107.1	50,888,881	50,888,902	46
aae-miR-33-5p	mature	NC_035107.1	50,888,926	50,888,946	28
aae-miR-2940	precursor	NC_035107.1	53,172,117	53,172,226	--
aae-miR-2940-5p	mature	NC_035107.1	53,172,121	53,172,143	14,190
aae-miR-2940-3p	mature	NC_035107.1	53,172,204	53,172,225	28,004
aae-miR-210	precursor	NC_035107.1	54,892,609	54,892,677	--
aae-miR-210-5p	mature	NC_035107.1	54,892,618	54,892,639	172
aae-miR-210-3p	mature	NC_035107.1	54,892,653	54,892,673	4,851
aae-miR-87	precursor	NC_035107.1	80,682,381	80,682,488	--
aae-miR-87-3p	mature	NC_035107.1	80,682,402	80,682,423	3,914
aae-miR-87-5p	mature	NC_035107.1	80,682,450	80,682,471	38
aae-miR-N014	precursor	NC_035107.1	100,807,223	100,807,328	--
aae-miR-N014-3p	mature	NC_035107.1	100,807,234	100,807,255	252
aae-miR-N014-5p	mature	NC_035107.1	100,807,296	100,807,318	--
aae-miR-927	precursor	NC_035107.1	115,022,922	115,022,998	--
aae-miR-927-5p	mature	NC_035107.1	115,022,930	115,022,951	5,358
aae-miR-927-3p	mature	NC_035107.1	115,022,972	115,022,993	180
aae-miR-981	precursor	NC_035107.1	118,556,333	118,556,424	--
aae-miR-981-5p	mature	NC_035107.1	118,556,349	118,556,370	16
aae-miR-981-3p	mature	NC_035107.1	118,556,391	118,556,412	6,540
aae-miR-N001	precursor	NC_035107.1	144,289,656	144,289,738	--
aae-miR-N001-5p	mature	NC_035107.1	144,289,669	144,289,689	302
aae-miR-970	precursor	NC_035107.1	157,238,399	157,238,507	--
aae-miR-970-5p	mature	NC_035107.1	157,238,424	157,238,446	--
aae-miR-970-3p	mature	NC_035107.1	157,238,464	157,238,484	2,236
aae-miR-252	precursor	NC_035107.1	175,818,151	175,818,222	--
aae-miR-252-3p	mature	NC_035107.1	175,818,158	175,818,179	144
aae-miR-252-5p	mature	NC_035107.1	175,818,191	175,818,212	7,206
aae-miR-929	precursor	NC_035107.1	198,495,510	198,495,598	--
aae-miR-929-5p	mature	NC_035107.1	198,495,527	198,495,547	68
aae-miR-929-3p	mature	NC_035107.1	198,495,565	198,495,585	7
aae-miR-279	precursor	NC_035107.1	207,007,771	207,007,866	--
aae-miR-279-5p	mature	NC_035107.1	207,007,789	207,007,814	18
aae-miR-279-3p	mature	NC_035107.1	207,007,832	207,007,851	17,987
aae-miR-996	precursor	NC_035107.1	207,012,520	207,012,618	--
aae-miR-996-5p	mature	NC_035107.1	207,012,537	207,012,555	--
aae-miR-996-3p	mature	NC_035107.1	207,012,584	207,012,603	297
aae-miR-11900	precursor	NC_035107.1	223,944,057	223,944,163	--
aae-miR-11900-5p	mature	NC_035107.1	223,944,071	223,944,095	--
aae-miR-11900-3p	mature	NC_035107.1	223,944,129	223,944,152	1,722
aae-miR-999	precursor	NC_035107.1	252,222,287	252,222,366	--
aae-miR-999-5p	mature	NC_035107.1	252,222,294	252,222,316	20
aae-miR-999-3p	mature	NC_035107.1	252,222,333	252,222,354	51,820
aae-miR-34	precursor	NC_035107.1	302,603,044	302,603,173	--
aae-miR-34-3p	mature	NC_035107.1	302,603,058	302,603,080	839

aae-miR-34-5p	mature	NC_035107.1	302,603,138	302,603,158	105,277
aae-miR-277	precursor	NC_035107.1	302,603,801	302,603,895	--
aae-miR-277-3p	mature	NC_035107.1	302,603,814	302,603,835	73,536
aae-miR-277-5p	mature	NC_035107.1	302,603,863	302,603,883	152
aae-miR-317	precursor	NC_035107.1	302,667,116	302,667,208	--
aae-miR-317-3p	mature	NC_035107.1	302,667,130	302,667,154	50,047
aae-miR-317-5p	mature	NC_035107.1	302,667,171	302,667,192	1,639
aae-miR-12	precursor	NC_035107.1	305,613,535	305,613,657	--
aae-miR-12-3p	mature	NC_035107.1	305,613,549	305,613,570	616
aae-miR-12-5p	mature	NC_035107.1	305,613,618	305,613,640	2,080
aae-miR-1889	precursor	NC_035107.1	305,613,942	305,614,045	--
aae-miR-1889-3p	mature	NC_035107.1	305,613,945	305,613,966	171
aae-miR-1889-5p	mature	NC_035107.1	305,614,020	305,614,041	6,907
aae-miR-283	precursor	NC_035107.1	305,625,581	305,625,684	--
aae-miR-283-3p	mature	NC_035107.1	305,625,601	305,625,622	--
aae-miR-283-5p	mature	NC_035107.1	305,625,647	305,625,669	19,871
aae-miR-1175	precursor	NC_035107.1	307,010,034	307,010,118	--
aae-miR-1175-3p	mature	NC_035107.1	307,010,048	307,010,068	3,686
aae-miR-1175-5p	mature	NC_035107.1	307,010,083	307,010,106	2,190
aae-miR-1174	precursor	NC_035107.1	307,010,195	307,010,318	--
aae-miR-1174-3p	mature	NC_035107.1	307,010,201	307,010,222	21,498
aae-miR-1174-5p	mature	NC_035107.1	307,010,287	307,010,309	150
aae-miR-iab_4	precursor	NC_035107.1	308,965,416	308,965,490	--
aae-miR-iab_4-3p	mature	NC_035107.1	308,965,424	308,965,447	--
aae-miR-iab_4-5p	mature	NC_035107.1	308,965,460	308,965,481	354
aae-miR-10	precursor	NC_035107.1	310,530,461	310,530,550	--
aae-miR-10-5p	mature	NC_035107.1	310,530,476	310,530,497	28,567
aae-miR-10-3p	mature	NC_035107.1	310,530,516	310,530,538	3,985
aae-miR-993	precursor	NC_035107.1	310,672,009	310,672,110	--
aae-miR-993-3p	mature	NC_035107.1	310,672,022	310,672,045	114
aae-miR-993-5p	mature	NC_035107.1	310,672,072	310,672,093	186
aae-miR-219	precursor	NC_035108.1	3,715,251	3,715,337	--
aae-miR-219-5p	mature	NC_035108.1	3,715,262	3,715,284	20
aae-miR-219-3p	mature	NC_035108.1	3,715,307	3,715,329	50
aae-miR-N013	precursor	NC_035108.1	30,841,052	30,841,132	--
aae-miR-N013-3p	mature	NC_035108.1	30,841,063	30,841,084	--
aae-miR-N013-5p	mature	NC_035108.1	30,841,100	30,841,122	--
aae-miR-11897a	precursor	NC_035108.1	46,313,953	46,314,028	--
aae-miR-11897a-5p	mature	NC_035108.1	46,313,964	46,313,985	--
aae-miR-11897a-3p	mature	NC_035108.1	46,313,997	46,314,018	--
aae-miR-190	precursor	NC_035108.1	48,811,855	48,811,956	--
aae-miR-190-3p	mature	NC_035108.1	48,811,879	48,811,900	28
aae-miR-190-5p	mature	NC_035108.1	48,811,920	48,811,942	8,942
aae-miR-11894b	precursor	NC_035108.1	58,783,349	58,783,409	--
aae-miR-11894b-3p	mature	NC_035108.1	58,783,351	58,783,372	--
aae-miR-11894b-5p	mature	NC_035108.1	58,783,388	58,783,409	9
aae-miR-11894a_5	precursor	NC_035108.1	58,785,157	58,785,217	--
aae-miR-11894a_5-3p	mature	NC_035108.1	58,785,159	58,785,180	--
aae-miR-11894a_5-5p	mature	NC_035108.1	58,785,196	58,785,217	--
aae-miR-11894a_4	precursor	NC_035108.1	58,785,849	58,785,909	--

aae-miR-11894a_4-3p	mature	NC_035108.1	58,785,851	58,785,872	--
aae-miR-11894a_4-5p	mature	NC_035108.1	58,785,888	58,785,909	--
aae-miR-11894a_3	precursor	NC_035108.1	58,786,283	58,786,343	--
aae-miR-11894a_3-3p	mature	NC_035108.1	58,786,285	58,786,306	--
aae-miR-11894a_3-5p	mature	NC_035108.1	58,786,322	58,786,343	--
aae-miR-11894_2	precursor	NC_035108.1	58,787,425	58,787,485	--
aae-miR-11894a_1	precursor	NC_035108.1	58,787,947	58,788,007	--
aae-miR-11894a_1-3p	mature	NC_035108.1	58,787,949	58,787,970	--
aae-miR-11894a_1-5p	mature	NC_035108.1	58,787,986	58,788,007	--
aae-miR-276a	precursor	NC_035108.1	73,120,439	73,120,530	--
aae-miR-276a-3p	mature	NC_035108.1	73,120,453	73,120,474	--
aae-miR-276a-5p	mature	NC_035108.1	73,120,495	73,120,514	209
aae-miR-276b	precursor	NC_035108.1	73,509,389	73,509,473	--
aae-miR-276b-3p	mature	NC_035108.1	73,509,400	73,509,421	7
aae-miR-276b-5p	mature	NC_035108.1	73,509,443	73,509,463	114
aae-miR-988	precursor	NC_035108.1	93,351,371	93,351,446	--
aae-miR-988-5p	mature	NC_035108.1	93,351,381	93,351,402	483
aae-miR-988-3p	mature	NC_035108.1	93,351,418	93,351,439	285
aae-miR-281	precursor	NC_035108.1	96,469,343	96,469,440	--
aae-miR-281-3p	mature	NC_035108.1	96,469,362	96,469,383	111,160
aae-miR-281-5p	mature	NC_035108.1	96,469,399	96,469,420	59,517
aae-miR-282	precursor	NC_035108.1	96,911,956	96,912,051	--
aae-miR-282-3p	mature	NC_035108.1	96,911,967	96,911,988	--
aae-miR-282-5p	mature	NC_035108.1	96,912,019	96,912,040	26
aae-miR-2b	precursor	NC_035108.1	107,235,872	107,235,959	--
aae-miR-2b-3p	mature	NC_035108.1	107,235,889	107,235,908	123
aae-miR-2b-5p	mature	NC_035108.1	107,235,924	107,235,946	136
aae-miR-2a	precursor	NC_035108.1	107,237,486	107,237,583	--
aae-miR-2a-3p	mature	NC_035108.1	107,237,506	107,237,525	7,324
aae-miR-2a-5p	mature	NC_035108.1	107,237,547	107,237,569	14
aae-miR-13	precursor	NC_035108.1	107,237,641	107,237,725	--
aae-miR-13-3p	mature	NC_035108.1	107,237,650	107,237,672	5,908
aae-miR-13-5p	mature	NC_035108.1	107,237,692	107,237,713	296
aae-miR-2c	precursor	NC_035108.1	107,237,990	107,238,068	--
aae-miR-2c-3p	mature	NC_035108.1	107,237,996	107,238,017	629
aae-miR-2c-5p	mature	NC_035108.1	107,238,041	107,238,061	--
aae-miR-71	precursor	NC_035108.1	107,238,282	107,238,359	--
aae-miR-71-3p	mature	NC_035108.1	107,238,288	107,238,309	929
aae-miR-71-5p	mature	NC_035108.1	107,238,333	107,238,354	2,315
aae-miR-9a	precursor	NC_035108.1	108,461,937	108,462,018	--
aae-miR-9a-3p	mature	NC_035108.1	108,461,988	108,462,009	95
aae-miR-263b	precursor	NC_035108.1	153,371,119	153,371,210	--
aae-miR-263b-3p	mature	NC_035108.1	153,371,132	153,371,153	7
aae-miR-263b-5p	mature	NC_035108.1	153,371,174	153,371,196	4,834
aae-miR-2943a	precursor	NC_035108.1	158,468,836	158,468,917	--
aae-miR-2943b	precursor	NC_035108.1	158,468,998	158,469,069	--
aae-miR-11899	precursor	NC_035108.1	182,319,033	182,319,115	--
aae-miR-11899-3p	mature	NC_035108.1	182,319,042	182,319,064	22
aae-miR-11899-5p	mature	NC_035108.1	182,319,083	182,319,104	22
aae-miR-137	precursor	NC_035108.1	189,612,044	189,612,148	--

aae-miR-137-5p	mature	NC_035108.1	189,612,068	189,612,090	--
aae-miR-137-3p	mature	NC_035108.1	189,612,107	189,612,128	2,746
aae-miR-7	precursor	NC_035108.1	222,802,529	222,802,618	--
aae-miR-7-5p	mature	NC_035108.1	222,802,545	222,802,568	55,960
aae-miR-7-3p	mature	NC_035108.1	222,802,585	222,802,606	12
aae-miR-932	precursor	NC_035108.1	231,381,139	231,381,234	--
aae-miR-932-5p	mature	NC_035108.1	231,381,157	231,381,179	1,464
aae-miR-932-3p	mature	NC_035108.1	231,381,195	231,381,216	253
aae-miR-11911	precursor	NC_035108.1	256,105,994	256,106,072	--
aae-miR-11911-3p	mature	NC_035108.1	256,106,006	256,106,027	31
aae-miR-11911-5p	mature	NC_035108.1	256,106,037	256,106,059	--
aae-miR-11893	precursor	NC_035108.1	257,889,031	257,889,109	--
aae-miR-11893-5p	mature	NC_035108.1	257,889,042	257,889,062	16
aae-miR-11893-3p	mature	NC_035108.1	257,889,078	257,889,099	160
aae-miR-2941-1	precursor	NC_035108.1	268,455,991	268,456,097	--
aae-miR-2941-1-3p	mature	NC_035108.1	268,456,011	268,456,033	--
aae-miR-2941-1-5p	mature	NC_035108.1	268,456,054	268,456,075	--
aae-miR-2941-2	precursor	NC_035108.1	268,456,300	268,456,395	--
aae-miR-2941-2-3p	mature	NC_035108.1	268,456,315	268,456,337	--
aae-miR-2941-2-5p	mature	NC_035108.1	268,456,357	268,456,376	--
aae-miR-2946	precursor	NC_035108.1	268,456,429	268,456,517	--
aae-miR-2946-3p	mature	NC_035108.1	268,456,442	268,456,463	2,650
aae-miR-2946-5p	mature	NC_035108.1	268,456,481	268,456,503	--
aae-miR-196	precursor	NC_035108.1	289,697,987	289,698,051	--
aae-miR-196-3p	mature	NC_035108.1	289,697,989	289,698,010	18
aae-miR-196-5p	mature	NC_035108.1	289,698,030	289,698,051	258
aae-miR-275	precursor	NC_035108.1	307,135,653	307,135,736	--
aae-miR-275-5p	mature	NC_035108.1	307,135,669	307,135,691	294
aae-miR-275-3p	mature	NC_035108.1	307,135,709	307,135,730	20,849
aae-miR-305	precursor	NC_035108.1	307,145,431	307,145,519	--
aae-miR-305-5p	mature	NC_035108.1	307,145,446	307,145,469	6,582
aae-miR-305-3p	mature	NC_035108.1	307,145,484	307,145,506	182
aae-miR-11895	precursor	NC_035108.1	324,949,484	324,949,564	--
aae-miR-11895-3p	mature	NC_035108.1	324,949,495	324,949,516	23
aae-miR-11895-5p	mature	NC_035108.1	324,949,533	324,949,554	579
aae-miR-133	precursor	NC_035108.1	330,417,047	330,417,166	--
aae-miR-133-5p	mature	NC_035108.1	330,417,066	330,417,088	--
aae-miR-133-3p	mature	NC_035108.1	330,417,128	330,417,149	1,927
aae-miR-N007	precursor	NC_035108.1	331,681,388	331,681,471	--
aae-miR-N007-5p	mature	NC_035108.1	331,681,399	331,681,422	--
aae-miR-N007-3p	mature	NC_035108.1	331,681,438	331,681,460	--
aae-miR-9b	precursor	NC_035108.1	335,780,561	335,780,653	--
aae-miR-9b-3p	mature	NC_035108.1	335,780,575	335,780,596	32
aae-miR-9b-5p	mature	NC_035108.1	335,780,616	335,780,639	15,091
aae-miR-79	precursor	NC_035108.1	335,780,865	335,780,961	--
aae-miR-79-3p	mature	NC_035108.1	335,780,885	335,780,907	33
aae-miR-79-5p	mature	NC_035108.1	335,780,924	335,780,946	21
aae-miR-306	precursor	NC_035108.1	335,781,035	335,781,164	--
aae-miR-306-3p	mature	NC_035108.1	335,781,050	335,781,071	23
aae-miR-306-5p	mature	NC_035108.1	335,781,125	335,781,145	9,248

aae-miR-9c	precursor	NC_035108.1	335,810,358	335,810,441	--
aae-miR-9c-3p	mature	NC_035108.1	335,810,365	335,810,386	679
aae-miR-9c-5p	mature	NC_035108.1	335,810,415	335,810,436	13,509
aae-miR-375	precursor	NC_035108.1	337,876,516	337,876,632	--
aae-miR-375-5p	mature	NC_035108.1	337,876,533	337,876,556	25
aae-miR-375-3p	mature	NC_035108.1	337,876,594	337,876,615	1,971
aae-miR-14	precursor	NC_035108.1	344,767,521	344,767,694	--
aae-miR-14-5p	mature	NC_035108.1	344,767,573	344,767,594	53
aae-miR-14-3p	mature	NC_035108.1	344,767,616	344,767,637	111,688
aae-miR-263a	precursor	NC_035108.1	348,784,426	348,784,523	--
aae-miR-263a-3p	mature	NC_035108.1	348,784,449	348,784,470	70
aae-miR-263a-5p	mature	NC_035108.1	348,784,488	348,784,510	7,142
aae-miR-1891	precursor	NC_035108.1	360,084,853	360,084,940	--
aae-miR-1891-3p	mature	NC_035108.1	360,084,868	360,084,889	1,816
aae-miR-1891-5p	mature	NC_035108.1	360,084,904	360,084,925	10,135
aae-miR-124	precursor	NC_035108.1	369,307,270	369,307,346	--
aae-miR-124-5p	mature	NC_035108.1	369,307,282	369,307,302	8
aae-miR-124-3p	mature	NC_035108.1	369,307,320	369,307,339	334
aae-miR-2945	precursor	NC_035108.1	374,947,133	374,947,215	--
aae-miR-2945-5p	mature	NC_035108.1	374,947,143	374,947,166	28
aae-miR-2945-3p	mature	NC_035108.1	374,947,185	374,947,205	28,652
aae-miR-100	precursor	NC_035108.1	375,117,321	375,117,447	--
aae-miR-100-5p	mature	NC_035108.1	375,117,352	375,117,373	122,990
aae-miR-100-3p	mature	NC_035108.1	375,117,392	375,117,413	738
aae-miR-let_7	precursor	NC_035108.1	375,131,491	375,131,564	--
aae-miR-let_7-5p	mature	NC_035108.1	375,131,498	375,131,518	29,246
aae-miR-let_7-3p	mature	NC_035108.1	375,131,539	375,131,560	--
aae-miR-125	precursor	NC_035108.1	375,131,761	375,131,856	--
aae-miR-125-5p	mature	NC_035108.1	375,131,782	375,131,803	11,130
aae-miR-125-3p	mature	NC_035108.1	375,131,820	375,131,840	116
aae-miR-1	precursor	NC_035108.1	399,501,016	399,501,092	--
aae-miR-1-3p	mature	NC_035108.1	399,501,024	399,501,045	185,601
aae-miR-1-5p	mature	NC_035108.1	399,501,063	399,501,084	57
aae-miR-309b	precursor	NC_035108.1	402,152,468	402,152,539	--
aae-miR-309b-3p	mature	NC_035108.1	402,152,473	402,152,493	--
aae-miR-2944a	precursor	NC_035108.1	402,152,656	402,152,718	--
aae-miR-2944a-3p	mature	NC_035108.1	402,152,661	402,152,680	--
aae-miR-2944a-5p	mature	NC_035108.1	402,152,693	402,152,714	14
aae-miR-2944b	precursor	NC_035108.1	402,152,793	402,152,855	--
aae-miR-2944b-3p	mature	NC_035108.1	402,152,795	402,152,818	9
aae-miR-2944b-5p	mature	NC_035108.1	402,152,831	402,152,852	--
aae-miR-286a	precursor	NC_035108.1	402,153,076	402,153,173	--
aae-miR-286a-3p	mature	NC_035108.1	402,153,090	402,153,111	--
aae-miR-286a-5p	mature	NC_035108.1	402,153,141	402,153,161	--
aae-miR-1890	precursor	NC_035108.1	413,654,953	413,655,039	--
aae-miR-1890-3p	mature	NC_035108.1	413,654,962	413,654,983	1,319
aae-miR-1890-5p	mature	NC_035108.1	413,655,002	413,655,023	19
aae-miR-184	precursor	NC_035108.1	417,599,368	417,599,456	--
aae-miR-184-3p	mature	NC_035108.1	417,599,381	417,599,402	69,644
aae-miR-184-5p	mature	NC_035108.1	417,599,420	417,599,441	33

aae-miR-965	precursor	NC_035108.1	429,778,740	429,778,819	--
aae-miR-965-5p	mature	NC_035108.1	429,778,750	429,778,773	111
aae-miR-965-3p	mature	NC_035108.1	429,778,792	429,778,813	98
aae-miR-N010	precursor	NC_035108.1	439,707,833	439,707,917	--
aae-miR-N010-3p	mature	NC_035108.1	439,707,839	439,707,861	--
aae-miR-N010-5p	mature	NC_035108.1	439,707,889	439,707,910	--
aae-miR-11905	precursor	NC_035108.1	459,139,427	459,139,496	--
aae-miR-11905-5p	mature	NC_035108.1	459,139,429	459,139,450	--
aae-miR-11905-3p	mature	NC_035108.1	459,139,474	459,139,496	--
aae-miR-980	precursor	NC_035109.1	1,054,518	1,054,594	--
aae-miR-980-3p	mature	NC_035109.1	1,054,524	1,054,545	22
aae-miR-980-5p	mature	NC_035109.1	1,054,562	1,054,585	58
aae-miR-315	precursor	NC_035109.1	3,444,752	3,444,844	--
aae-miR-315-5p	mature	NC_035109.1	3,444,768	3,444,790	1,412
aae-miR-315-3p	mature	NC_035109.1	3,444,810	3,444,831	--
aae-miR-2796	precursor	NC_035109.1	6,069,674	6,069,755	--
aae-miR-2796-3p	mature	NC_035109.1	6,069,686	6,069,708	6,503
aae-miR-2796-5p	mature	NC_035109.1	6,069,724	6,069,745	--
aae-miR-1000	precursor	NC_035109.1	74,204,044	74,204,141	--
aae-miR-1000-3p	mature	NC_035109.1	74,204,062	74,204,083	25
aae-miR-1000-5p	mature	NC_035109.1	74,204,099	74,204,119	471
aae-miR-998	precursor	NC_035109.1	75,489,576	75,489,668	--
aae-miR-998-3p	mature	NC_035109.1	75,489,590	75,489,610	6,521
aae-miR-998-5p	mature	NC_035109.1	75,489,632	75,489,652	79
aae-miR-11	precursor	NC_035109.1	75,489,838	75,489,952	--
aae-miR-11-3p	mature	NC_035109.1	75,489,865	75,489,886	109,070
aae-miR-11-5p	mature	NC_035109.1	75,489,905	75,489,928	1,272
aae-miR-971	precursor	NC_035109.1	82,678,939	82,679,027	--
aae-miR-971-5p	mature	NC_035109.1	82,678,954	82,678,975	--
aae-miR-971-3p	mature	NC_035109.1	82,678,994	82,679,016	342
aae-miR-92b	precursor	NC_035109.1	93,418,505	93,418,588	--
aae-miR-92b-3p	mature	NC_035109.1	93,418,516	93,418,537	1,124
aae-miR-92b-5p	mature	NC_035109.1	93,418,555	93,418,577	46
aae-miR-92a	precursor	NC_035109.1	93,477,587	93,477,669	--
aae-miR-92a-3p	mature	NC_035109.1	93,477,597	93,477,618	684
aae-miR-92a-5p	mature	NC_035109.1	93,477,637	93,477,658	18
aae-miR-2765	precursor	NC_035109.1	101,895,441	101,895,528	--
aae-miR-2765-3p	mature	NC_035109.1	101,895,455	101,895,477	--
aae-miR-2765-5p	mature	NC_035109.1	101,895,491	101,895,512	94
aae-miR-31	precursor	NC_035109.1	136,174,140	136,174,218	--
aae-miR-31-5p	mature	NC_035109.1	136,174,151	136,174,171	2,812
aae-miR-31-3p	mature	NC_035109.1	136,174,186	136,174,206	218
aae-miR-bantam	precursor	NC_035109.1	178,082,826	178,082,897	--
aae-miR-bantam-5p	mature	NC_035109.1	178,082,831	178,082,854	3,881
aae-miR-bantam-3p	mature	NC_035109.1	178,082,873	178,082,895	118,306
aae-miR-N011	precursor	NC_035109.1	240,317,264	240,317,340	--
aae-miR-N011-5p	mature	NC_035109.1	240,317,272	240,317,292	--
aae-miR-N011-3p	mature	NC_035109.1	240,317,315	240,317,335	--
aae-miR-278	precursor	NC_035109.1	243,123,879	243,123,960	--
aae-miR-278-3p	mature	NC_035109.1	243,123,890	243,123,910	11,340

aae-miR-278-5p	mature	NC_035109.1	243,123,930	243,123,952	77
aae-miR-307	precursor	NC_035109.1	244,794,355	244,794,446	--
aae-miR-307-3p	mature	NC_035109.1	244,794,413	244,794,433	62
aae-miR-11898a	precursor	NC_035109.1	276,079,731	276,079,789	--
aae-miR-11898a-5p	mature	NC_035109.1	276,079,733	276,079,753	--
aae-miR-11898a-3p	mature	NC_035109.1	276,079,769	276,079,789	--
aae-miR-309a	precursor	NC_035109.1	309,335,445	309,335,537	--
aae-miR-309a-3p	mature	NC_035109.1	309,335,453	309,335,473	44
aae-miR-309a-5p	mature	NC_035109.1	309,335,497	309,335,520	--
aae-miR-286b	precursor	NC_035109.1	309,335,988	309,336,085	--
aae-miR-286b-3p	mature	NC_035109.1	309,335,998	309,336,020	101
aae-miR-286b-5p	mature	NC_035109.1	309,336,056	309,336,076	--
aae-miR-316	precursor	NC_035109.1	311,919,545	311,919,636	--
aae-miR-316-5p	mature	NC_035109.1	311,919,564	311,919,585	2,537
aae-miR-316-3p	mature	NC_035109.1	311,919,605	311,919,626	--
aae-miR-10365	precursor	NC_035109.1	320,775,611	320,775,704	--
aae-miR-10365-3p	mature	NC_035109.1	320,775,622	320,775,643	67
aae-miR-10365-5p	mature	NC_035109.1	320,775,673	320,775,694	1,248
aae-miR-8	precursor	NC_035109.1	328,527,946	328,528,025	--
aae-miR-8-5p	mature	NC_035109.1	328,527,956	328,527,977	5,071
aae-miR-8-3p	mature	NC_035109.1	328,527,995	328,528,017	293,522
aae-miR-193	precursor	NC_035109.1	360,501,785	360,501,871	--
aae-miR-193-5p	mature	NC_035109.1	360,501,796	360,501,817	43
aae-miR-193-3p	mature	NC_035109.1	360,501,839	360,501,860	11
aae-miR-989	precursor	NC_035109.1	366,439,055	366,439,170	--
aae-miR-989-3p	mature	NC_035109.1	366,439,082	366,439,103	71,200
aae-miR-989-5p	mature	NC_035109.1	366,439,124	366,439,147	108
aae-miR-11898b	precursor	NC_035109.1	381,026,876	381,026,934	--
aae-miR-11898b-5p	mature	NC_035109.1	381,026,878	381,026,898	--
aae-miR-11898b-3p	mature	NC_035109.1	381,026,914	381,026,934	--
aae-miR-2942	precursor	NC_035109.1	390,116,452	390,116,534	--
aae-miR-2942-3p	mature	NC_035109.1	390,116,460	390,116,482	34
aae-miR-2942-5p	mature	NC_035109.1	390,116,497	390,116,525	--
aae-miR-957	precursor	NC_035109.1	398,466,618	398,466,696	--
aae-miR-957-5p	mature	NC_035109.1	398,466,625	398,466,647	--
aae-miR-957-3p	mature	NC_035109.1	398,466,670	398,466,691	6,360
aae-miR-308	precursor	NC_035109.1	403,338,480	403,338,556	--
aae-miR-308-5p	mature	NC_035109.1	403,338,525	403,338,546	1,736
aae-miR-285	precursor	NC_035109.1	406,875,020	406,875,124	--
aae-miR-285-3p	mature	NC_035109.1	406,875,047	406,875,068	3,050
aae-miR-285-5p	mature	NC_035109.1	406,875,086	406,875,108	38

Table S4. Validation of small RNA-Seq using RT-qPCR for nine miRNAs using 5s ribosomal RNA as reference. Ct, cycle threshold. FC, fold-change. CPM, counts per million.

miR	sample	Ct Tet 5s	Ct wAlbB 5s	Log_2FC 5s	CPM Tet RNA-Seq	CPM wAlbB RNA-Seq	Log_2FC RNA-Seq
miR-184-3p	2a	0.009	0.012	0.457	30086.72	21683.43	-0.473
miR-184-3p	2b	0.016	0.018	0.145	23221.31	24299.58	0.065
miR-184-3p	2c	0.010	0.015	0.630	22508.70	21626.34	-0.058
miR-184-3p	6a	0.014	0.019	0.390	32567.13	19805.09	-0.718
miR-184-3p	6b	0.015	0.036	1.230	33438.72	19300.74	-0.793
miR-184-3p	6c	0.007	0.016	1.192	32832.30	21851.47	-0.587
miR-184-3p	12a	0.017	0.029	0.752	24933.55	34228.03	0.457
miR-184-3p	12b	0.012	0.018	0.655	33762.11	30179.70	-0.162
miR-184-3p	12c	0.016	0.032	0.944	26798.87	33585.99	0.326
miR-190-5p	2a	0.000	0.000	2.636	2509.97	3615.57	0.527
miR-190-5p	2b	0.000	0.000	1.443	3101.17	3461.22	0.158
miR-190-5p	2c	0.000	0.000	2.134	1212.08	3742.09	1.626
miR-190-5p	6a	0.000	0.000	1.104	3096.70	4766.74	0.622
miR-190-5p	6b	0.000	0.000	2.837	1374.58	5014.42	1.867
miR-190-5p	6c	0.000	0.000	1.134	3392.17	3950.37	0.220
miR-190-5p	12a	0.000	0.000	1.961	4055.78	3533.31	-0.199
miR-190-5p	12b	0.000	0.000	0.970	3555.78	3889.45	0.129
miR-190-5p	12c	0.000	0.000	2.704	3862.88	3848.92	-0.005
miR-276b-5p	2a	0.000	0.000	1.516	40.87	72.63	0.829
miR-276b-5p	2b	0.000	0.001	1.210	58.17	85.84	0.561
miR-276b-5p	2c	0.000	0.001	2.921	37.08	82.97	1.162
miR-276b-5p	6a	0.000	0.000	2.975	63.86	87.03	0.447
miR-276b-5p	6b	0.000	0.001	2.337	53.96	74.04	0.456
miR-276b-5p	6c	0.000	0.001	1.468	77.81	97.04	0.319
miR-276b-5p	12a	0.000	0.000	-0.488	103.90	111.71	0.105
miR-276b-5p	12b	0.000	0.000	1.339	95.74	98.63	0.043
miR-276b-5p	12c	0.000	0.000	1.719	100.45	124.16	0.306
miR-2941-1-3p	2a	0.001	0.000	-2.837	20.22	11.85	-0.771
miR-2941-1-3p	2b	0.003	0.000	-3.179	14.02	8.95	-0.647
miR-2941-1-3p	2c	0.002	0.000	-2.631	13.69	9.04	-0.599
miR-2941-1-3p	6a	0.002	0.002	-0.478	7.69	6.97	-0.141
miR-2941-1-3p	6b	0.003	0.002	-0.945	8.04	10.64	0.405
miR-2941-1-3p	6c	0.003	0.002	-0.411	6.31	10.21	0.695
miR-2941-1-3p	12a	0.002	0.003	0.400	6.51	15.04	1.208
miR-2941-1-3p	12b	0.002	0.003	0.464	3.96	8.73	1.142
miR-2941-1-3p	12c	0.004	0.003	-0.610	3.21	11.68	1.863
miR-308-5p	2a	0.000	0.000	0.261	575.30	438.89	-0.390
miR-308-5p	2b	0.001	0.001	0.153	476.21	439.99	-0.114
miR-308-5p	2c	0.000	0.001	0.752	585.22	397.62	-0.558
miR-308-5p	6a	0.001	0.001	0.692	829.02	530.99	-0.643
miR-308-5p	6b	0.001	0.001	0.616	912.30	435.70	-1.066
miR-308-5p	6c	0.001	0.001	0.694	790.31	510.73	-0.630
miR-308-5p	12a	0.001	0.002	0.872	860.03	670.63	-0.359
miR-308-5p	12b	0.001	0.001	0.490	954.22	806.46	-0.243
miR-308-5p	12c	0.001	0.001	0.507	986.13	853.10	-0.209

miR-31-3p	2a	0.004	0.007	0.801	43.95	123.44	1.490
miR-31-3p	2b	0.004	0.013	1.557	92.16	107.76	0.226
miR-31-3p	2c	0.003	0.012	1.872	76.43	121.31	0.666
miR-31-3p	6a	0.001	0.001	0.350	57.95	80.32	0.471
miR-31-3p	6b	0.002	0.002	-0.052	65.44	88.08	0.429
miR-31-3p	6c	0.001	0.001	0.047	82.44	89.51	0.119
miR-31-3p	12a	0.002	0.001	-0.955	82.19	69.96	-0.232
miR-31-3p	12b	0.001	0.001	0.024	94.16	77.97	-0.272
miR-31-3p	12c	0.002	0.001	-0.370	88.52	68.35	-0.373
miR-34-5p	2a	0.013	0.007	-0.849	27140.77	21783.18	-0.317
miR-34-5p	2b	0.023	0.009	-1.323	16827.98	17866.52	0.086
miR-34-5p	2c	0.018	0.010	-0.867	25961.26	16777.92	-0.630
miR-34-5p	6a	0.028	0.037	0.433	44573.74	44187.88	-0.013
miR-34-5p	6b	0.036	0.033	-0.097	46879.10	39614.15	-0.243
miR-34-5p	6c	0.021	0.039	0.930	41125.76	45430.29	0.144
miR-34-5p	12a	0.042	0.054	0.376	55620.93	60299.06	0.117
miR-34-5p	12b	0.027	0.033	0.274	53802.74	62360.43	0.213
miR-34-5p	12c	0.050	0.065	0.387	49636.71	61823.21	0.317
miR-2940-5p	2a	0.013	0.018	0.436	6635.10	9981.70	0.589
miR-2940-5p	2b	0.014	0.032	1.146	5879.61	6367.91	0.115
miR-2940-5p	2c	0.007	0.032	2.205	5552.17	7372.72	0.409
miR-2940-5p	6a	0.005	0.029	2.447	9381.17	12281.40	0.389
miR-2940-5p	6b	0.007	0.050	2.789	10162.76	17800.88	0.809
miR-2940-5p	6c	0.005	0.053	3.284	7853.51	12732.24	0.697
miR-2940-5p	12a	0.020	0.007	-1.498	12546.02	11703.10	-0.100
miR-2940-5p	12b	0.011	0.027	1.313	11820.16	19155.17	0.696
miR-2940-5p	12c	0.010	0.018	0.826	11481.30	13675.19	0.252
miR-309a-3p	2a	0.000	0.000	-0.909	7.47	3.74	-0.998
miR-309a-3p	2b	0.000	0.000	-2.181	8.41	2.16	-1.960
miR-309a-3p	2c	0.000	0.000	0.084	11.41	3.01	-1.921
miR-309a-3p	6a	0.000	0.000	0.646	14.78	20.66	0.483
miR-309a-3p	6b	0.000	0.000	0.770	17.22	20.85	0.276
miR-309a-3p	6c	0.000	0.000	-0.643	13.46	20.43	0.602
miR-309a-3p	12a	0.000	0.000	0.134	19.23	32.35	0.750
miR-309a-3p	12b	0.000	0.000	0.372	20.97	38.40	0.873
miR-309a-3p	12c	0.000	0.001	0.842	22.02	28.55	0.375
miR-2946-3p	2a	0.001	0.000	-2.927	506.30	640.88	0.340
miR-2946-3p	2b	0.016	0.001	-4.772	780.72	510.69	-0.612
miR-2946-3p	2c	0.003	0.001	-2.273	375.89	541.12	0.526
miR-2946-3p	6a	0.006	0.008	0.557	613.78	1235.53	1.009
miR-2946-3p	6b	0.014	0.021	0.623	477.58	1286.26	1.429
miR-2946-3p	6c	0.019	0.017	-0.188	990.94	1168.23	0.237
miR-2946-3p	12a	0.016	0.030	0.846	1123.97	1613.20	0.521
miR-2946-3p	12b	0.013	0.024	0.885	1613.31	1573.64	-0.036
miR-2946-3p	12c	0.021	0.041	0.974	1336.55	1802.25	0.431

Table S5. Peaks in coverage of small RNA-Seq reads aligned to the *w*AlbB NZ_CP031221.1. ‘w2’, ‘w6’, ‘w12’ refer to WB2 2 dpe, WB2 6 dpe, and WB2 12 dpe, respectively. Two intergenic regions containing peaks 1 and 9 showed a pattern of read-coverage reminiscent of precursor miRNA loci, and were predicted to form stem-loop structures (Figs. S3-5A).

Name	timepoint(s) in which peak was detected	Start	End	Length	Gene feature overlapping with peak
Peak_1	w2_w6_12	75270	75320	50	Intergenic
Peak_2	w2	366284	366294	10	gene-DEJ70_RS01810
Peak_3	w2_w6_12	435829	435894	65	gene-DEJ70_RS02130;Name=ssrS
Peak_4	w2	501208	501226	18	gene-DEJ70_RS02470
Peak_5	w2_w6	547309	547328	19	gene-DEJ70_RS02685
Peak_6	w2_w6_12	561122	561143	21	gene-DEJ70_RS02745
Peak_7	w2_w6	713377	713397	20	gene-DEJ70_RS03405;Name=typA
Peak_8	w2_w6_12	752990	753012	22	gene-DEJ70_RS03590;product=tRNA-Phe
Peak_9	w2_w6_12	783582	783604	22	Intergenic
Peak_10	w2_w6_12	961047	961066	19	gene-DEJ70_RS04590;product=tRNA-Leu
Peak_11	w2	963346	963361	15	gene-DEJ70_RS04600;pseudogene
Peak_12	w12	1008958	1008977	19	gene-DEJ70_RS04850
Peak_13	w2_w6_12	1043770	1043789	19	gene-DEJ70_RS05085;Name=rplB
Peak_14	w12	1068991	1069008	17	rna-DEJ70_RS05225;product=5S ribosomal RNA
Peak_15	w2_w6	1144140	1144159	19	gene-DEJ70_RS05535;Name=putA
Peak_16	w2_w6_12	1337501	1337520	19	Intergenic
Peak_17	w2_w6_12	1367049	1367068	19	Intergenic
Peak_18	w2_w6_12	1476626	1476645	19	gene-DEJ70_RS07100
